# Large‐scale proteomic approach to stable isotope labeling kinetics (SILK) for studying protein turnover in blood and cerebrospinal fluid in Alzheimer’s disease

**DOI:** 10.1002/alz.091534

**Published:** 2025-01-03

**Authors:** Sylvain Lehmann

**Affiliations:** ^1^ Université de Montpellier, Montpellier France

## Abstract

**Background:**

Protein metabolism and turnover can be monitored using tracer methods, notably stable isotope labeling kinetics (SILK) based on 13C‐leucine incorporation. This approach has been used in Alzheimer’s disease, specifically analyzing the turnover in cerebrospinal fluid of biomarkers of interest, including amyloid peptides, leading to major pathophysiological insights (Nature medicine 12:856‐861). This was achieved using immunoprecipitation mass spectrometry, which enables to track a small number of targets present in low concentration. Complementarily, we have recently validated a new large‐scale SILK proteomics workflow and bioinformatics pipeline capable of assessing the turnover of hundreds of proteins (Analytical chemistry 91:15500‐15508). Here, we use this workflow on SILK blood and CSF samples to generate a comprehensive model of protein dynamics that can potentially be used to compare samples generated in amyloid‐positive and amyloid‐negative patients.

**Methods:**

We analyzed blood and CSF samples collected for up to 36 hours, every 3 hours, after a 9‐hour infusion of 13C‐leucine. After depletion of major proteins, samples were reduced, alkylated and digested before C18 fractionation. LC‐MS acquisitions were performed on Evosep coupled to TIMS TOF HT (Bruker Daltonics). Data acquisition generated a list of peptides uploaded and process on skyline. We used a Bayesian hierarchical model to provide robust and comprehensive data of protein turnover (doi.org/10.1101/2023.10.30.564713).

**Result:**

In a first series of analyses CSF MS data covered 3,156 proteins and 22,842 distinct peptides, of which 16,913 contained at least one leucine. 2,417 distinct peptides usable for dynamics modelling determined the dynamics of 869 proteins in CSF. In plasma, the same process led to 1,260 proteins covered by 9,243 peptides, 6,788 of which contained at least one leucine, enabling the dynamics of 271 proteins in plasma to be estimated. The number of proteins detected with dynamics data in both plasma and CSF was 194. We report an accurate 3‐biological compartment model able simultaneously account for the protein dynamics observed in plasma and the CSF including a hidden central nervous system compartment.

**Conclusion:**

The models developed and optimized for 3‐biological‐compartment system has now been implemented and will be applied to a SILK cohort generated in amyloid‐positive and amyloid‐negative patients.

